# Skin microbiota and diabetic foot ulcers

**DOI:** 10.3389/fmicb.2025.1575081

**Published:** 2025-07-01

**Authors:** Jiaqi Lou, Ziyi Xiang, Xiaoyu Zhu, Jiliang Li, Guoying Jin, Shengyong Cui, Neng Huang, Xin Le, Youfen Fan, Qionghui Sun

**Affiliations:** ^1^Burn Department, Ningbo No.2 Hospital, Ningbo, China; ^2^Institute of Pathology, Faculty of Medicine, University of Bonn, Bonn, Germany; ^3^Health Science Center, Ningbo University, Ningbo, China

**Keywords:** skin microbiota, diabetic foot ulcers, *Staphylococcus*, *Pseudomonas*, microorganisms

## Abstract

Skin microbiota is the microbial population on the skin surface, which has a symbiotic relationship with the host skin and plays an important role in maintaining skin health and regulating immune responses. In patients with diabetic foot ulcers (DFUs), the skin microbiota is unbalanced. The abundance of pathogenic bacteria such as *Staphylococcus aureus* and *Pseudomonas aeruginosa* increases, forming biofilms, destroying the skin barrier function, aggravating infection, and leading to poor wound healing. Studies have shown that the diversity of skin microbiota is positively correlated with the severity of ulcers, and regulating its composition and function may be an important strategy to improve DFUs healing. In recent years, with the development of molecular biology technology, progress has been made in the study of skin microbiota, such as 16S rRNA gene sequencing technology to understand its composition changes and explore the interaction mechanism with the host immune system. Based on this, some new therapeutic approaches are being explored, such as the use of probiotics or antibacterial drugs to modulate the composition of the microbiota and the development of microbiota-based personalized treatment regimens. However, there are still challenges in current research. For example, the composition and function of skin microbiota are affected by many factors, and there are relatively few studies on other microorganisms such as fungi and viruses. In the future, it is necessary to further explore its diversity and the interaction mechanism with the host, and develop more effective treatment methods to improve the prognosis of patients with DFUs.

## 1 Overview of the skin microbiota

The skin microbiome refers to the diverse microbial communities that inhabit the skin surface, including bacteria, fungi, and viruses. These microorganisms engage in complex interactions with the host skin and together constitute the skin microbiome. The composition of the skin microbiota varies among individuals due to genetic, environmental, and lifestyle factors. Studies have shown that skin microbiota plays a crucial role in maintaining skin health, regulating immune responses, and resisting pathogen invasion (Grice and Segre, 2011). For example, microbiota produce antimicrobial peptides (Kloepper et al., 2008) and regulate immune cell activity to defend against pathogens. In addition, it is involved in skin metabolic processes and affects skin barrier function.

In diabetic foot ulcers (DFUs), dysbiosis of the skin microbiota—defined as a disruption in the balance of microbial communities, often characterized by reduced diversity, overgrowth of pathogenic bacteria, and loss of commensal species—is associated with impaired wound healing. This imbalance can lead to reduced antimicrobial peptide production, altered immune regulation, and compromised skin barrier function, all of which contribute to chronic inflammation and delayed tissue repair. Recent studies (Johannes and Römer, 2010) have shown that the composition and function of the skin microbiota are influenced not only by host genetic and environmental factors, but also by the immune status of the skin. The diversity of skin microbiota is closely related to skin health, and commensal bacteria promote normal skin barrier function by regulating the immune microenvironment. Notably, the composition of the skin microbiota varies significantly across body sites and between individuals. For example, moist areas such as the cubital fossa are usually rich in *Staphylococcus* and *Corynebacterium*, while dry areas such as the forearm and leg harbor more diverse bacterial communities (Costello et al., 2009). In addition, the composition of the microbiota can be influenced by age, gender, and ethnicity (Zhang et al., 2021).

## 2 Symbiotic relationship between skin and microbiota

There is a close symbiotic relationship between the skin and its microbiota. The skin provides an ideal environment for microbial growth, providing temperature, humidity, and nutrients. In return, microbes contribute to normal skin physiology through their metabolic activity and immunomodulatory effects. For example, certain commensal bacteria metabolize amino acids, steroids, fatty acids, and sugars on the skin surface (Melnyk et al., 2022), thereby affecting the skin microenvironment. Certain commensal bacteria, including *Staphylococcus epidermidis* and *Cutibacterium acnes*, enhance skin health through antimicrobial peptide production (e.g., bacteriocins) and immunomodulation (Costello et al., 2009). For example, *S. epidermidis* activates TLR4 signaling to recruit immune cells, while *C. acnes* metabolites regulate T-cell responses via the AhR pathway (Zhang et al., 2021; Melnyk et al., 2022). The skin microbiota also enhances skin resistance to pathogens by modulating the host immune system. This symbiotic balance is essential for skin health, and its disruption may lead to skin diseases such as eczema and psoriasis (Timm et al., 2020). Recent study (Yun et al., 2021) has shown that specific microbial communities, such as *Pseudoalteromonas luteoviolacea* and *Shewanella colwelliana*, play an important role in fish wound healing by regulating inflammation and promoting cell proliferation. Similarly, in humans, the diversity of the skin microbiota is critical for wound healing, and alterations in microbial composition significantly affect the speed and quality of wound repair. The microbial community of DFU is mainly composed of Gram-positive and Gram-negative bacteria, including *Staphylococcus aureus*, *Streptococcus* spp., *Pseudomonas aeruginosa* and *Enterobacteriaceae*, etc. (Maity et al., 2024). These microorganisms delay ulcer healing by forming biofilms and increasing antibiotic resistance (Dinić et al., 2024). In DFUs, the imbalance of skin microbiota not only affects wound healing, but also exacerbates inflammation by modulating the host immune response. For example, *Staphylococcus aureus* in DFUs can secrete toxins and enzymes that disrupt the skin barrier and form biofilms that resist host immune defenses and antibiotic treatment. Studies have also shown that patients with DFU have a lower diversity of skin microbial communities and a higher abundance of pathogenic bacteria, which may lead to difficult ulcer healing (MacDonald et al., 2019). Specifically, high abundance of *Staphylococcus aureus* and *Pseudomonas* is strongly associated with chronicity of ulcers and increased risk of infection (Dhankhar et al., 2024).

Obesity is associated with DFUs through multiple mechanisms. First, inflammation linked to obesity could help explain the high incidence of DFUs in obese patients. Chronic low-grade inflammation in adipose tissue, driven by macrophage infiltration and the release of pro-inflammatory cytokines (e.g., TNF-α, IL-6), may contribute to systemic immune dysregulation and impaired wound healing (Johannes and Römer, 2010). Second, the overall incidence of T2DM is higher in obese persons, and T2DM is a major risk factor for DFUs due to neuropathy, peripheral vascular disease, and impaired immune function (Costello et al., 2009). Third, obese individuals may have more skin folds that create moist and dark environments, which encourage the growth of problematic microbes. These microenvironments favor the proliferation of pathogenic bacteria, further exacerbating DFU development and worsening outcomes (Zhang et al., 2021; Sechovcová et al., 2024). Therefore, modulation of the composition and function of the skin microbiota, along with addressing obesity-related inflammation and metabolic dysregulation, may be key strategies to improve DFU healing (Norton et al., 2024).

## 3 Diabetic foot ulcers and their pathogenesis

**Figure 1:**
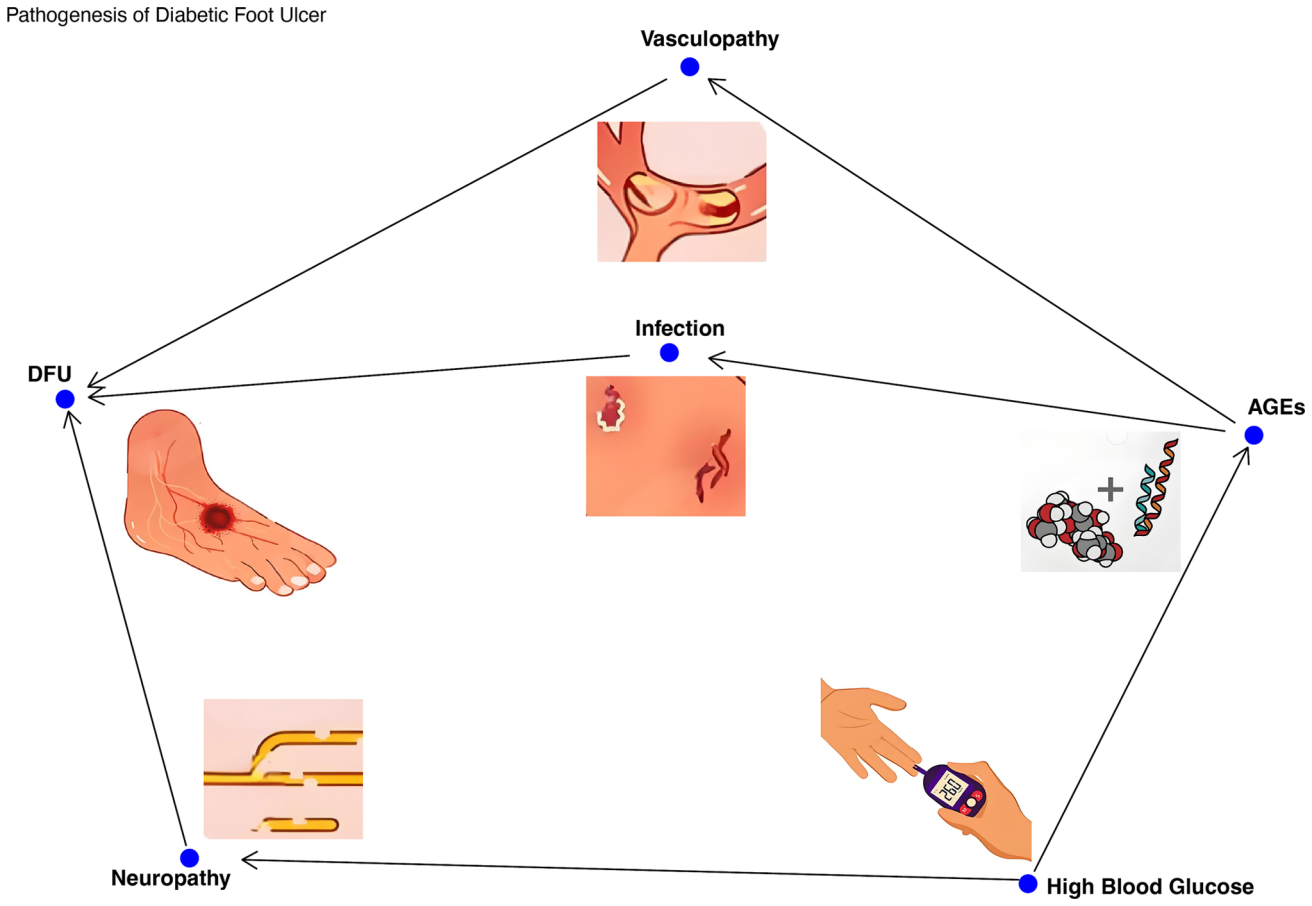
The pathogenesis of foot ulcer in diabetes. The multifactorial pathogenesis of DFUs was demonstrated, including hyperglycemia, neuropathy, vascular disease, impaired immune function, and microbial imbalance. Arrow: Indicates a causal relationship, such as high blood sugar leading to neuropathy, neuropathy causing insensitivity to foot trauma, and ultimately leading to ulcer formation.

DFUs is a common complication in patients with diabetes mellitus, which seriously affects the quality of life and health. The pathogenesis of DFUs is multifactorial, involving neuropathy, vasculopathy, and infection due to hyperglycemia (Makrantonaki et al., 2025). When blood glucose is too high, sugars combine with proteins to produce advanced glycation end products (AGEs) (MacDonald et al., 2019; Makrantonaki et al., 2025). These substances will disrupt the balance of cell proliferation and apoptosis and the interaction between intracellular and extracellular environment, and further aggravate tissue damage. Nerve damage caused by hyperglycemia reduces sensitivity to foot trauma and pressure (Jan et al., 2024) and manifests as sensory neuropathy, motor neuropathy, and autonomic neuropathy. These neuropathies can lead to the loss of pain protection of the foot, micro-tears due to stress changes, and the development of ulcers over time, making the patient prone to ulcers. In addition, diabetes-related vascular lesions reduce blood flow in the foot, and peripheral vascular lesions mostly occur in microvessels at the end of the limbs, which are mainly related to oxidative stress, non-enzymatic glycosylation and other factors caused by metabolic disorders (Makrantonaki et al., 2025). These factors can lead to vascular endothelial damage, which in turn causes tissue hypoxia, resulting in reduced secretion of vasodilators, leading to ischemia and hindering ulcer healing (Jan et al., 2024; Nazari et al., 2025). Patients with DFU often exhibit impaired immune function, which hinders pathogen clearance and exacerbates ulcer severity. Studies (Yun et al., 2021) have shown that patients with DFU have reduced diversity of the microbiota and increased abundance of pathogens such as *Staphylococcus aureus* and *Pseudomonas aeruginosa*. These pathogens form biofilms that increase the persistence of infection and therapeutic challenges, while inhibiting the growth of commensal bacteria and further disrupting the balance of the microbiota. Infection is also one of the important causes of diabetic foot ulcers. Bacterial secretions and extracellular matrix form a stubborn and low-permeability polysacpatient-protein complex, that is, bacterial biofilm (BBF), which seriously affects ulcer healing and is prone to drug resistance (Qin et al., 2024). Obesity is closely related to the development and progression of DFUs (Sechovcová et al., 2024). Obese individuals often have a chronic inflammatory state, which exacerbates the pathological process of DFUs. Immune dysregulation caused by obesity, such as increased secretion of proinflammatory cytokines (for example, IL-6, TNF-α) by monocytes, also indirectly affects the skin immune response (Sechovcová et al., 2024; Li et al., 2024), making DFU patients more susceptible to infection (Figure 1).

The above content has primarily focused on the host pathophysiology underlying the development of DFUs. The following section will delve into the specific roles and mechanisms of the skin microbiota in DFU progression.

## 4 Role of skin microbiota in DFUs

The skin microbiota plays an important role in the development and progression of DFUs. It is worth noting that even without the presence of an ulcer, the skin of diabetic patients may show an overall change in the *flora.* Compared with healthy individuals, patients with DFU exhibit different microbiota composition, and the skin microbial community of diabetic foot ulcers is mainly composed of bacteria, fungi, and viruses (Zhang et al., 2023). Among them, bacteria are the most important components, and common bacteria include *Staphylococcus aureus*, *Staphylococcus epidermidis*, *Pseudomonas aeruginosa* and *Cutibacterium acnes* (Huang et al., 2025). The relative abundance and diversity of these bacteria in diabetic foot ulcers are significantly different from those in healthy skin. The abundance of *S. aureus* in diabetic foot ulcers is significantly higher than that in healthy skin, while the abundance of *Staphylococcus epidermidis* and *Propionobacter acnes* is relatively low (Zubair and Ahmad, 2019; Wang et al., 2021). These commensal bacteria often have anti-inflammatory and antibacterial properties, regulate immune responses and promote wound healing. Skin of patients with diabetic foot ulcers also lacks some beneficial Gram-negative bacteria, such as *Roseomonas mucosa* (Liu et al., 2022), which secrete antimicrobial peptides and sphingolipid metabolites that inhibit the growth of *Staphylococcus aureus*, thus protecting the skin from pathogens (Zubair and Ahmad, 2019; Liu et al., 2022). There is also an increase in the proportion of anaerobic bacteria in DFUs, which are able to inhibit the growth of beneficial bacteria by producing harmful metabolites (Cheong et al., 2022), thus further disrupting the balance of skin microorganisms, such as *Anaerococcus* spp. and *Porphyromonas* spp., et al., these anaerobic bacteria also play an important role in the chronic process of ulcers (Gjødsbøl et al., 2013; Smith et al., 2016). Therefore, the imbalance of skin microbiota in DFU patients may be a key factor leading to impaired wound healing.

However, the formation of an ulcer can further exacerbate and precipitate more significant changes in the skin flora. Biofilms are the main organizational form of microorganisms in diabetic foot ulcers, consisting of a complex microbial community of bacteria and fungi surrounded by a polymer matrix composed of polysaccharides, lipids, proteins, and nucleic acids (Yang et al., 2024). Studies have shown that the existence rate of biofilm in chronic wounds is as high as 78.2%, which is significantly higher than that in acute wounds (Sen et al., 2020). Biofilm formation not only increases microbial resistance, but also leads to delayed wound healing by inhibiting the host immune response and interfering with the normal healing process (Yang et al., 2024). Studies (Di Perri and Ferlazzo, 2022; Fleming et al., 2022) have shown that microorganisms in biofilms can form a protective layer by secreting exopolysaccharide and other substances, thereby escaping the immune surveillance of the host.

Recent studies have further elucidated the complex interactions between the skin microbiota and DFUs, which, in general, both promote and inhibit wound healing (Huang et al., 2025; Cheong et al., 2022). During the healing process of DFU, microorganisms inhibit wound healing through a variety of mechanisms. First, microbes can delay healing by producing toxins and enzymes (Radzieta et al., 2022), damaging host tissues. The large presence of *S. aureus* and other pathogenic bacteria in diabetic foot ulcers will trigger a strong inflammatory response and release a variety of inflammatory mediators, such as interleukin-6 (IL-6) and tumor necrosis factor-α (TNF-α) (Zhang et al., 2024), which will further aggravate tissue damage and delay the healing process. In addition, *S. aureus* biofilm can also enhance antibiotic resistance, which further hinders wound healing. Metabolites of Gram-negative bacteria such as *Pseudomonas aeruginosa* and *Escherichia coli* can activate the Toll-like receptor (TLR) signaling pathway (Zhen et al., 2024), leading to the release of inflammatory factors, thereby aggravating the inflammatory response. Some skin commensal bacteria can also promote the expression of metalloproteinases (MMPs), leading to excessive degradation of extracellular matrix (ECM), thereby hindering ulcer healing (Jiang et al., 2024). This promotion of MMP expression not only prevents the healing of existing ulcers by degrading the ECM, but may also contribute to the occurrence of new ulcers by compromising the structural integrity of healthy skin. Microbial metabolites in DFUs can also trigger oxidative stress, leading to the increase of intracellular reactive oxygen species (ROS) levels (Gao et al., 2024). Oxidative stress can not only damage DNA, proteins and lipids of cells, but also inhibit cell proliferation and migration (Walton et al., 2019), and further delay the ulcer healing process. The microorganisms in DFUs are not isolated. The coexistence and interaction of multiple microorganisms in diabetic foot ulcers have an important impact on the healing process. For example, studies have found that *Staphylococcus aureus* and *Pseudomonas aeruginosa* have synergistic effects between *Staphylococcus aureus* and *Pseudomonas aeruginosa* in co-infected wounds (Malekian et al., 2019), which can enhance each other’s pathogenicity and biofilm formation ability. Thus, the infection and inflammatory response of ulcers are further aggravated. Although the growth of *Pseudomonas aeruginosa* is observed to be inhibited to a certain extent, its presence increases the expression of virulence factors of *Staphylococcus aureus*, leading to further delay of wound healing (Malekian et al., 2019; Di Domenico et al., 2020). In addition, the abundance of facultative anaerobes such as *Enterobacter* is significantly associated with wound nonhealing, which may be more advantageous in the healing process due to their stronger adaptability in different metabolic environments (Islam et al., 2025).

On the other hand, microbes can influence the healing process by interacting with the host immune system and modulating the inflammatory response. Pathogens such as *Staphylococcus aureus* and *Pseudomonas aeruginosa* can escape the host immune surveillance and even survive in the host cells (Malekian et al., 2019; Di Domenico et al., 2020; Agwu et al., 2010). In addition, antimicrobial molecules such as Perforin-2 play a key role in the clearance of intracellular pathogens, but pathogens such as *Staphylococcus aureus* can inhibit the expression of Perforin-2, thus persisting in the wound and hindering healing (Pastar et al., 2021). On the contrary, in terms of promoting wound healing, certain bacteria in the skin microbial community, such as *S. epidermidis*, can inhibit the growth of *S. aureus* and reduce inflammatory response by secreting antimicrobial peptides (AMPs) (Tan et al., 2022). In addition, *S. epidermidis* can also activate Langerhans cells in the skin (Chimento et al., 2017), promote the recruitment and activation of immune cells, or activate the host innate immune response and promote healing through TLR4 receptor-mediated signaling pathway (Zhen et al., 2024). Certain beneficial bacteria, such as *Propionate acnes* and *Staphylococcus epidermis*, can activate the AhR signaling pathway and promote the generation of regulatory T cells (Tregs) by producing metabolites such as short-chain fatty acids (SCFAs), thereby inhibiting excessive inflammatory responses (Tindjau et al., 2024; Hansson and Faergemann, 1995) and promoting wound healing. For example, propionic acid produced by *Propionibacterium acnes* can activate the AhR signaling pathway and promote the generation of Tregs, thereby inhibiting the inflammatory response and accelerating wound healing (Tindjau et al., 2024). *Staphylococcus aureus* promotes glutamine metabolism in keratinocytes by inducing hypoxia-inducible factor-1α (HIF-1α) signaling pathway (Wang et al., 2021), thereby increasing the production of interleukin-1β (IL-1β) (Cao et al., 2024) and promoting hair follicle regeneration (Wickersham et al., 2017). This metabolic regulation not only enhances the tolerance of stem cells to injury, but also improves the regenerative capacity.

Some bacterial metabolites can also directly promote tissue repair. For example, lipopeptides secreted by *S. epidermidis* can activate the β-catenin signaling pathway, inhibit skin inflammation, and promote the synthesis and deposition of collagen, thereby accelerating wound healing (Li et al., 2019). In addition, *S. epidermidis* can also promote the proliferation and migration of skin cells by secreting growth factors (Zielińska et al., 2023), which further promotes wound healing. Liu et al. (2022) identified a strain of *Staphylococcus epidermidis* with potent and broad-spectrum activity against Gram-positive pathogens, mediated by the bacteriocin micrococcin P1 (MP1). This strain was found to reduce *Staphylococcus aureus* infection and accelerate the closure of *Staphylococcus aureus* infected wounds. In addition, *Roseomonas mucosa* is able to secrete sphingolipid metabolites, such as sphingosine-1-phosphate (S1P) (Doudi et al., 2024), which have antibacterial and anti-inflammatory effects and can promote the recovery of skin barrier function and angiogenesis (Chen et al., 2023). The potential of using these beneficial strains for a “probiotic” approach to DFUs highlights the therapeutic potential of harnessing the skin microbiota.

The use of nanoparticle strategies to deliver antimicrobial substances such as MP1 (Liu et al., 2022) has shown promise to overcome the limitations of natural substances, further highlighting the potential of microbiota transplantation therapy in DFUs. Reduced microbiota diversity in DFU patients is positively correlated with ulcer severity, underscoring the importance of microbiota diversity in maintaining skin health and promoting wound healing. Changes in the composition of the microbiota also significantly affect the speed and quality of DFUs wound healing. Studies have found that the diversity of skin microorganisms is positively correlated with the healing speed of diabetic foot ulcers. High diversity of skin microbial communities can better resist the invasion of pathogenic bacteria, reduce the formation of biofilm, and thus promote wound healing (Gimblet et al., 2017). In addition, the composition of skin microbes is also closely related to the healing stage of diabetic foot ulcers. In the early stage of healing, the abundance of pathogenic bacteria such as *Staphylococcus aureus* is high, while in the late stage of healing, the abundance of beneficial bacteria such as lactic acid bacteria and *Propionibacterium acnes* gradually increases (Sikorska and Smoragiewicz, 2013).

In addition, angiogenesis is also one of the key processes in DFU healing. Studies (Argañaraz Aybar et al., 2022; Okonkwo and DiPietro, 2017) have shown that the function of vascular endothelial cells is impaired in the skin of patients with diabetic foot ulcers, as indicated by reduced angiogenesis. This is closely related to skin microbial imbalance, especially the large presence of *Staphylococcus aureus* can inhibit the proliferation and migration of vascular endothelial cells, leading to the block of angiogenesis. Another important aspect is the influence of skin microbiota on the biomechanical properties of scars, which is also relevant to the healing process of DFUs. Jung et al. (2025) have shown that the skin microbiota can influence the biomechanical properties of burn scars and that specific microorganisms are associated with changes in scar elasticity and extensibility. This suggests that the skin microbiota may also play a role in the biomechanical properties of DFUs scars, possibly affecting the long-term outcome of DFUs healing (Table 1).

**Table 1:** Microbial influence on healing of diabetic foot ulcers.

Microbial species	Positive effects	Negative effects
Bacteria		
*Staphylococcus aureus*	Induction of HIF-1α signaling in keratinocytes promotes glutamine metabolism, increases IL-1β production, and promotes hair follicle regeneration	Produces toxins and enzymes that damage host tissues and delay healing; Biofilms enhance antibiotic resistance and hinder healing
*Staphylococcus epidermidis*	Secretes AMPs to inhibit the growth of *S. aureus* and reduce inflammatory response; Activates Langerhans cells in the skin and promotes the recruitment and activation of immune cells via TLR4 receptor-mediated signaling pathways	When co-infected with *S. aureus*, enhances the pathogenicity and biofilm formation ability of *S. aureus*, aggravating infection and inflammatory response
*Pseudomonas aeruginosa*	Not mentioned	Activates TLR signaling pathway, leading to the release of inflammatory factors and aggravating the inflammatory response; Synergistic effect with *S. aureus* enhances pathogenicity and biofilm formation ability
*Cutibacterium acnes*	Produces SCFAs to activate AhR signaling pathway and promote the generation of Tregs, inhibiting excessive inflammatory response	When co-infected with *S. aureus*, may affect wound healing
*Roseomonas mucosa*	Secretes sphingolipid metabolites (e.g., S1P) with antibacterial and anti-inflammatory effects, promoting skin barrier function recovery and angiogenesis	Not mentioned
*Lactobacillus* spp.	Secretes antimicrobial peptides and metabolites to inhibit pathogenic bacteria growth, reduce inflammatory response, and promote wound healing	Not mentioned
*Enterococcus* spp.	Not mentioned	Forms biofilms and increases resistance to host immune response, delaying wound healing
*Corynebacterium* spp.	Not mentioned	Forms biofilms and increases resistance to host immune response, delaying wound healing
*Granulicatella* spp.	Not mentioned	Forms biofilms and increases resistance to host immune response, delaying wound healing
*Streptococcus pneumoniae*	Not mentioned	Forms biofilms and increases resistance to host immune response, delaying wound healing
*Streptococcus pyogenes*	Not mentioned	Forms biofilms and increases resistance to host immune response, delaying wound healing
*Klebsiella* spp.	Not mentioned	Forms biofilms and increases resistance to host immune response, delaying wound healing
*Proteus* spp.	Not mentioned	Forms biofilms and increases resistance to host immune response, delaying wound healing

## 5 Recent advances and innovations

With the development of molecular biology technology, significant progress has been made in the study of skin microbiota. For example, 16S rRNA gene sequencing has allowed us to gain a more comprehensive understanding of the composition and changes of the microbiota in DFUs (Kozich et al., 2013). When performing 16S rRNA gene sequencing, it is crucial to consider the selection of primer regions, with the V3-V4 region being commonly used for skin microbiota studies as it provides good coverage and resolution. Bioinformatics pipelines typically involve quality filtering, operational taxonomic unit (OTU) picking, and taxonomic classification. Alpha diversity metrics such as Shannon and Simpson indices are used to assess microbial diversity within samples, while beta diversity metrics like Bray–Curtis dissimilarity are employed to analyze differences between samples. For detailed protocols, the study by Kozich et al. (2013) provides a comprehensive guide that many researchers follow. In addition, studies have explored the interaction between skin microbiota and the host immune system, revealing that certain commensal bacteria can enhance the skin’s antimicrobial defense by regulating immune cell activity (Zou et al., 2020; Travis et al., 2020).

### 5.1 Based-skin microbiota therapeutic approaches

Although antibiotics are the traditional method for the treatment of diabetic foot ulcer infection, their effect is limited and it is easy to lead to the generation of drug-resistant bacteria. In recent years, researchers have begun to explore new antimicrobial strategies, such as targeted biofilm therapy, phage therapy, and microbial community transplantation. Therapeutic strategies targeting biofilms, such as the use of biofilm dispersants or antimicrobial peptides, can effectively destroy biofilms, thereby improving the efficacy of antibiotics (Kao and Fritz, 2025). Phage therapy restores the balance of the microbial community and promotes wound healing by using specific phages to target and kill pathogenic bacteria, such as *S. aureus*, without affecting other beneficial bacteria. Microbial community transplantation is to restore the normal microbial community structure by transplanting healthy microbial community to the site of diabetic foot ulcer, thereby promoting wound healing. However, it is important to note that ulcers are open wounds and even healthy or normal flora should not be colonizing open skin and could contribute to disease once barrier function is lost. Therefore, the idea of microbial transplantation is not to directly colonize the open wounds, but rather to modulate the skin microbiota before an ulcer develops. By restoring a balanced microbial community, we can enhance the skin’s barrier function and reduce the risk of ulcer formation.

In terms of novel therapeutic approaches based on skin microbiota studies, the use of probiotics or antimicrobial agents to modulate microbiota composition to promote DFU healing is under investigation. Probiotics have the ability to promote wound healing through mechanisms such as the competitive exclusion of pathogens, the secretion of antimicrobial substances, and the modulation of immune responses (Lou et al., 2024). For example, lactic acid bacteria can produce lactic acid and short-chain fatty acids, which not only have antibacterial effects, but also can regulate the local pH value and inhibit the growth of pathogens (Wu et al., 2025). It has also been found that some bacteria can secrete phenolic soluble peptides (PSMs) (Cogen et al., 2010; Chen et al., 2024), which can regulate the immune response and metabolic state of host cells. Promote wound healing. In addition, probiotics are also able to accelerate wound healing by inducing macrophage polarization to M2 type and promoting anti-inflammatory response and angiogenesis (Wang et al., 2025). In addition, there is evidence to suggest that a healthy gut microbiota may alter several other processes that protect other parts of the body from unfavorable colonization. For instance, the gut microbiota can influence the production of various immune cells and antibodies, which circulate throughout the body and help maintain the balance of microbiota on the skin and other surfaces (Lou et al., 2024). The gut microbiota can also affect the production of short-chain fatty acids (SCFAs), which have anti-inflammatory effects and may indirectly protect the skin from harmful bacterial colonization (Tindjau et al., 2024; Hansson and Faergemann, 1995). However, further research is needed to fully understand these complex interactions and their therapeutic potential. The use of antimicrobial peptides and metabolites secreted by some beneficial strains can effectively inhibit the growth of *Staphylococcus aureus*, promote the recovery of skin barrier function and angiogenesis, and thus accelerate the healing of diabetic foot ulcers (Clausen and Agner, 2016). Plant derived compounds, such as Ganoderma spore oil, have also shown potential to accelerate burn wound healing by modulating microbiota and immune responses (Jiao et al., 2022). Finally, some studies (Meenakshi and Santhanakumar, 2023) have shown that oral probiotics can promote the healing of diabetic foot ulcers by regulating the intestinal microbial community and indirectly affecting the skin microbial community. For example, one study (Karimi et al., 2024) found that oral *Lactobacillus* can significantly reduce the ulcer area and reduce the level of inflammatory indicators in patients with diabetic foot ulcers.

Recent studies have also shown that nucleotide-binding oligomerization domain 2(NOD2) gene expression can regulate microbiota composition, thereby affecting wound healing (Perez-Chanona et al., 2014). In the NOD2 knockout mouse model, reduced microbiota diversity and increased abundance of *Pseudomonas aeruginosa* were observed, leading to delayed wound healing (Williams et al., 2017). The expression of NOD2 is significantly increased in patients with diabetic foot ulcers, and the activation of NOD2 will further aggravate the inflammatory response and inhibit the migration and proliferation of keratinocytes, thereby delaying healing (Cao et al., 2024). In addition, microbial infection induces the expression of antimicrobial peptides, such as mouse β-defensin 14 (mBD14), which, although antibacterial, may also delay healing further by affecting keratinocyte function (Miani et al., 2018). Microbiome technology enables us to more comprehensively understand the dynamic changes of skin microbiota, which provides the possibility to develop personalized treatment strategies based on microbiota characteristics. For example, specific microorganisms such as *Enterobacteriaceae* have been identified to be significantly associated with nonhealing chronic wounds, providing a basis for the development of targeted therapies (Finley et al., 2015). In addition, biofilm is a protective structure formed by bacteria in diabetic foot ulcers, which makes bacteria more resistant to antibiotics. In recent years, researchers have developed a series of biofilm inhibitors, such as silver ions and nanoparticles (Hu et al., 2024), which can destroy the structure of biofilm and enhance the bactericidal effect of antibiotics, thereby promoting wound healing.

## 6 Challenges and future directions

Despite remarkable progress in the study of skin microbiota, several challenges remain. The composition and function of the skin microbiota are influenced by multiple factors, including individual differences, environmental exposures, and lifestyle habits, which adds to the complexity of the study. Current studies mainly focus on bacterial communities, with limited exploration of fungi, viruses and other microbial taxa. Future studies must delve into the diversity of the skin microbiota and its interactions with the host to develop more effective therapeutic strategies to modulate the microbiota and promote DFU healing. In addition, future studies should focus on the dynamics of the skin microbiota and its role in different disease states. Longitudinal studies that track the temporal dynamics of skin microbiota in DFU patients are essential to elucidate the relationship between microbial changes and wound healing outcomes. Future studies should also consider individual differences such as microbiota composition and function in relation to age, sex, and ethnicity. In addition, monitoring the composition and diversity of skin microorganisms can predict the prognosis of wound healing and provide a reference for clinical treatment (Gomes et al., 2022). In the realm of 16S rRNA gene sequencing technology, advancements such as the use of PacBio and Oxford Nanopore (Ashari et al., 2025) technologies offer higher taxonomic resolution, enabling more accurate identification of microbial species. However, these technologies also present challenges, including higher costs and the need for advanced bioinformatics tools for data analysis. Overcoming these challenges and optimizing sequencing methods will be crucial for maximizing the benefits of 16S rRNA gene sequencing in microbiome research. Moreover, the integration of AI (Carrieri et al., 2021) and machine learning with large-scale microbiome data holds the potential to uncover new insights into disease mechanisms and therapeutic targets. This interdisciplinary approach could pave the way for more personalized and effective treatments for DFUs and other microbiome-related conditions.

### 6.1 Translation challenges in clinical practice

Despite the promising insights from skin microbiota research, several translational challenges hinder the clinical application of these findings. One major challenge is the colonization efficiency of probiotics. Studies such as Karimi et al. (2024) have shown that the ability of probiotics to colonize and exert their effects on DFU wounds is influenced by various factors including wound pH, temperature, and the presence of competing microorganisms. Another significant barrier is host-microbiome variability. The skin microbiota composition varies greatly among individuals due to genetic, environmental, and lifestyle factors. This variability makes it difficult to develop standardized probiotic or microbiota-based therapies that are effective across different patient populations. Recent clinical trials have highlighted the need for personalized approaches. Recent clinical trials have highlighted the potential of innovative therapeutic approaches. For example, the study by Schindler et al. (2024) investigated a novel gene therapy approach using a genetically modified lactic acid bacterium, demonstrated dosedependent efficacy, with 60% of patients in Cohort 4 achieving complete wound closure by end-of-treatment, and 83.3% within six months.

## 7 Conclusion

The skin microbiome plays a key role in the pathogenesis, progression, and healing of diabetic foot ulcers. Modulation of the composition and function of the skin microbiota is expected to develop new therapeutic strategies for DFUs. Future studies must further elucidate the regulatory mechanisms of the skin microbiota and its interaction with the host immune system to develop more effective therapies. The importance of microbiota diversity and its role in immune regulation for DFU healing cannot be ignored. Therefore, future studies should focus on exploring the mechanisms of microbiota regulation and its therapeutic potential in DFUs. In addition, the development of microbiota-based personalized treatment strategies should be prioritized to better meet the diverse needs of patients.
