# Correction: Skin microbiota and diabetic foot ulcers

**DOI:** 10.3389/fmicb.2025.1662172

**Published:** 2025-08-07

**Authors:** Jiaqi Lou, Ziyi Xiang, Xiaoyu Zhu, Jiliang Li, Guoying Jin, Shengyong Cui, Neng Huang, Xin Le, Youfen Fan, Qionghui Sun

**Affiliations:** ^1^Burn Department, Ningbo No.2 Hospital, Ningbo, China; ^2^Institute of Pathology, Faculty of Medicine, University of Bonn, Bonn, Germany; ^3^Health Science Center, Ningbo University, Ningbo, China

**Keywords:** skin microbiota, diabetic foot ulcers, *Staphylococcus*, *Pseudomonas*, microorganisms

A correction was made to the following sentence in Section 6.1, “Translation challenges in clinical practice,” due to factual inaccuracies concerning the cited study:

“For instance, a trial by Schindler et al. (2024) demonstrated that a one-size-fits-all probiotic regimen was effective in only 30% of DFU patients, underscoring the importance of tailoring treatments to individual microbiome profiles.”

The Schindler et al. (2024) study tested a genetically modified lactic acid bacterium producing human therapeutic proteins (a gene therapy product), not a probiotic regimen. The authors incorrectly label it as a “probiotic.” The claim of “30% efficacy” ignores the dose-dependent outcomes reported in the original study: Cohort 4 (high dose) achieved 60% wound closure by end-of-treatment and 83.3% at 6 months. Lower efficacy in Cohorts 1–3 was due to insufficient dosing, not lack of personalization. The Schindler study did not investigate microbiome profiles or personalized treatments. The authors erroneously used it to argue for microbiota-based personalization, which misrepresents the study's focus on gene therapy. The cited study explores gene therapy for wound healing, not probiotic efficacy or microbiome modulation. Citing it in the context of microbiota challenges is scientifically inappropriate.

The corrected text appears below:

“Recent clinical trials have highlighted the potential of innovative therapeutic approaches. For example, the study by Schindler et al. (2024) investigated a novel gene therapy approach using a genetically modified lactic acid bacterium, demonstrated dose-dependent efficacy, with 60% of patients in Cohort 4 achieving complete wound closure by end-of-treatment, and 83.3% within six months.”

The original version of this article has been updated.

